# First concrete documentation for presence of *Aedes (Stegomyia) albopictus* in Bolivia: dispelling previous anecdotes

**DOI:** 10.1590/0074-02760240063

**Published:** 2024-10-14

**Authors:** Frédéric Lardeux, Philippe Boussès, Rosenka Tejerina-Lardeux, Audric Berger, Christian Barnabé, Lineth Garcia

**Affiliations:** 1 Institut de Recherche pour le Développement, Unité Mixte de Recherche Maladies Infectieuses et Vecteurs: Ecologie, Génétique, Evolution et Contrôle, Montpellier, France; 2 Universidad Mayor de San Simón, Facultad de Medicina Dr Aurelio Meleán, Instituto de Investigaciones Biomédicas e Investigación Social, Cochabamba, Bolivia; 3 Institut de Recherche pour le Développement, Unité Mixte de Recherche Interactions Hôtes-Vecteurs-Parasites-Environnement dans les Maladies Tropicales Négligées dues aux Trypanosomatidés, Montpellier, France; 4 Universidad Mayor de San Simón, Facultad de Ciencias y Tecnología, Carrera de Biología, Laboratorio de Entomología Medica, Cochabamba, Bolivia

**Keywords:** Aedes albopictus, Bolivia, presence, identification, invasive species

## Abstract

**BACKGROUND:**

The presence of *Aedes albopictus* in Bolivia has been a subject of controversy, with a lack of concrete documentation.

**OBJECTIVES:**

This study aimed to provide evidence of *Ae. albopictus* presence in Bolivia.

**METHODS:**

Larval habitats were sampled in Rosario del Yata and San Agustín, Guayaramerín Municipality, Beni Department, northern Bolivia. Collected mosquito larvae were reared to the L4 and adult stages for morphological identification, with some specimens sequenced for confirmation.

**FINDINGS:**

*Aedes albopictus* was identified in multiple larval habitats within peridomestic areas, such as buckets, canisters, and cut plastic bottles used as flower vases in both localities, confirming its establishment in the area. This represents the first concrete documentation of the species in Bolivia. The collections (larvae and adults) have been deposited in the Medical Entomology Laboratory of the Universidad Mayor de San Simón in Cochabamba, Bolivia, and the Laboratory of Entomology of the Instituto Nacional de Laboratorios de Salud of the Ministry of Health in La Paz, Bolivia.

**MAIN CONCLUSION:**

Given its role as a vector for arboviruses such as dengue and Chikungunya, *Ae. albopictus* should be incorporated into the Bolivian National Programme of Vector Control for monitoring.

*Aedes (Stegomyia) albopictus* (Skuse, 1894) (Diptera: Culicidae) is regarded by the Invasive Species Specialist Group (ISSG) as one of the top 100 invasive species^1^ and is certainly the most invasive mosquito in the world. Commonly known as the “tiger mosquito” and originally from the tropical forests of Southeast Asia, it has undergone a rapid global expansion over the past four decades, driven by human activities.^2^ The initial sighting outside its original distribution range occurred in 1979 in Albania,^3^ and it has now established a presence on all continents, except Antarctica.^4^

In Latin America, *Ae. albopictus*, following its initial discovery in Brazil in 1986,^5^ was subsequently documented in Mexico in 1988. Between 1993 and 1998, its presence extended to the Dominican Republic, Cuba, Guatemala, the Cayman Islands, Colombia, and Argentina. Throughout the 2000s, the mosquito further spread to Canada, Bermuda, Trinidad and Tobago, Panama, Nicaragua, Costa Rica, Uruguay, Venezuela, Belize, and Haiti. More recent reports indicate its identification in Ecuador in 2017 and Jamaica in 2018.^6^

Bolivia, located in central South America, is a landlocked country bordered by Brazil to the north and east, Paraguay and Argentina to the south, Chile to the southwest, and Peru to the northwest. While Brazil and Argentina have documented the presence of *Ae. albopictus* within their territories, there is currently no evidence of the mosquito being reported in Chile and Peru.^6^ In Paraguay, the species has likely been found in various departments, including one bordering Bolivia (Boquerón),^7^ albeit never officially published in a scientific article.^6^ In Argentina, *Ae. albopictus* has been identified exclusively in the two provinces of Misiones and Corrientes, situated in the northwest of the country, bordering Paraguay. Notably, there is a considerable distance from the Bolivian border, approximately 1000 km, and no records of its presence have been reported in the intervening regions.^8^ In Brazil, the states bordering Bolivia, specifically Rondônia, Mato Grosso, and Mato Grosso do Sul, reported the presence of *Ae. albopictus* between 1991 and 2002.^9^ More recent observations confirming the presence of the mosquito have come from Acre State in 2022, specifically in the city of Rio Branco, located approximately 70 km in a straight line from the Bolivian border.^10^

The presence of *Ae. albopictus* in Bolivia has been a subject of controversy. Initial scientific reports were solely based on personal communications, particularly from former Pan American Health Organisation (PAHO) officials who held responsibilities in Bolivia before the 2000s.^11^ ^,^ ^12^ These reports indicated the species’ presence in the country since 1997 or potentially slightly earlier,^13^ lacking further details. However, this presence was not confirmed in the subsequent PAHO report.^14^ Nevertheless, these personal communications stem from an anecdote recounted to one of the authors of this article (FL) which mentioned that in the 1990s, *Ae. albopictus* was reportedly collected in Cotoca in the Santa Cruz Department without specifying whether “Cotoca” referred to the Botanical Garden of the city of Santa Cruz or a locality within the Department. The sample was reportedly sent to a laboratory in Argentina for species identification confirmation, yet neither the specific laboratory nor the contact scientist was disclosed. Although the identification was purportedly set to be confirmed, the sample was regrettably misplaced, and all subsequent efforts to locate it have proven futile, casting doubts on the actual existence of the collected specimens. Recently, in March 2023, a team of entomologists based in Santa Cruz de la Sierra claimed to have discovered the species in four locations within the municipality of San Ignacio de Velasco (Santa Cruz Department, Province of José Miguel de Velasco) (Lat. -16.38, Long. -60.9) as reported in a local press article^15^ and via social networks.^16^ Unfortunately, as far as we are aware, none of the leading entomological laboratories in Bolivia, including those in La Paz (INLASA) and Cochabamba (LEMUMSS), as well as the Ministry of Health, have received samples for confirmation, despite their persistent requests. Once again, legitimate doubts persist regarding the existence of *Ae. albopictus* specimens and/or their accurate identification.

The present article details the first documented report of *Ae. albopictus* in two localities in northern Bolivia, Rosario del Yata and San Agustín, within the Beni Department (Vaca Diez Province, Guayaramerin Municipality).

## MATERIALS AND METHODS

**Fig. 1:**
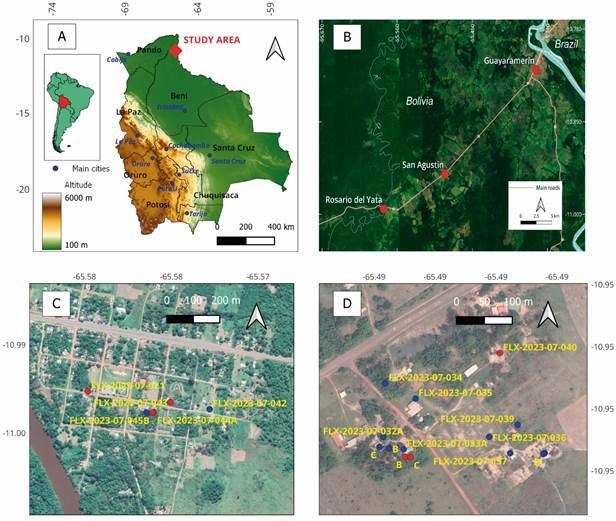
location of the study area within Bolivia, situated in the Amazonian region near the border with Brazil (A). Study area: localisation of the two localities, Rosario del Yata and San Agustín, situated along the main road from Guayaramerin (bordering Brazil) to Riberalta, in the Bolivian Amazonian region (B). Localisation of the sampling points in Rosario del Yata (C). Localisation of the sampling points in San Agustín (D) (red dots indicate *Aedes albopictus* positive habitats).

**Fig. 2:**
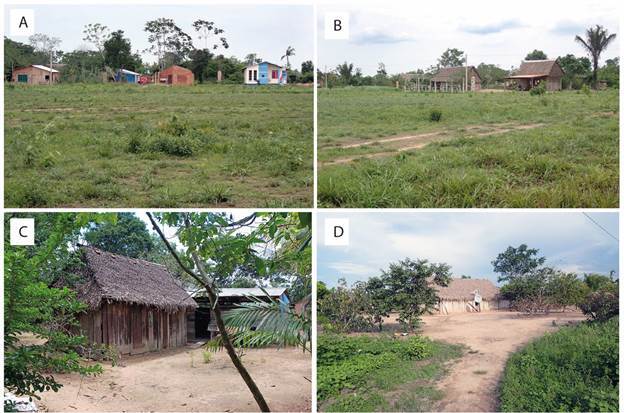
general view of human dwellings in Rosario del Yata (A, C) and San Agustín (B, D) depicting the dispersed habitat amidst a tree-lined environment.

*Study area* - The study area is located in the northeast of Bolivia, approximately 30-40 km west of Guayaramerín, a major city bordering Brazil (Fig. 1A). Sampling was conducted in two small localities, Rosario del Yata (lat. -10.99, long. -65.58) and San Agustín (lat. -10.95, long. -65.49), with San Agustín situated roughly 10 km east of Rosario del Yata (Fig. 1B). Both localities are situated along the main road between the two major cities, Guayaramerin and Riberalta, and are in close proximity to the Amazonian Forest that borders the road. Rosario del Yata is home to approximately 700 inhabitants residing in 180 houses, while San Agustín accommodates around 70 inhabitants in 15 houses. Both localities can be considered as rural. Houses are modest, lacking fences for separation. The backyards are kept relatively neat and include water wells. Due to the tropical climate, people engage in domestic activities outside their houses, often leaving small containers like buckets, pans, and pots outdoors, which serve as potential habitats for mosquito larvae. Domestic animals primarily include dogs, ducks, and chickens, provided water through waterers generally made from halved tyres cut longitudinally. Rainwater is also collected from roofs in 200 L drums or other small containers. Due to proximity to the Amazonian Forest, houses are amidst a tree-lined environment with trees that offer shade (Fig. 2A, B, C, D). Throughout the year, temperatures generally range from 20ºC to 35ºC, rarely dropping below 17ºC or rising above 38ºC. The hot season lasts from August to October, with September being the warmest month, featuring high temperatures of 35ºC and lows of 23ºC. The cool season lasts 6.6 months from December to June, with average daily high temperatures below 30ºC. June is the coldest month, with lows of 21ºC and highs of 29ºC. The wet season lasts about seven months from October to April, and the dry season about five months from May to September. February is the wettest month, with 232 mm of rain, while July is the driest, with 12 mm. The topography is relatively flat, with elevations ranging from 100 to 200 metres above sea level. The area surrounding the studied localities is covered by grassland, cropland, trees, and shrubs.

*Collection of mosquitoes and study area* - *Aedes albopictus* specimens were collected during a larger entomological survey conducted from November 12 to December 4, 2023 as part of the VECTOBOL initiative (https://vectobol.ird.fr/), jointly organised by Institut de Recherche pour le Développement (IRD, France) and Instituto de Investigaciones Biomédicas e Investigación Social (IIBISMED, Bolivia). The surveys involved collecting mosquito larvae from various types of larval habitats using dipping. In Rosario del Yata and San Agustín, mosquito larval habitats in the peridomestic areas primarily comprised artificial containers such as 200 L drums to collect rainwater from roofs, old tyres used for domestic animal waterers, used plastic bottles, buckets and all sorts of discarded small containers.

The first discovery of *Ae. albopictus* occurred in Rosario del Yata when a 200 L plastic container collecting rainwater in the peridomestic area of a house was sampled on November 23, 2023 (sample ID: FLX-2023-021 of Table). The collection mainly consisted of numerous small larvae resembling the genus “*Aedes*” and some *Toxorhynchites* sp. To precisely identify the species, larvae were reared to the L4 and adult stages. Subsequently, the first *Ae. albopictus* adults were morphologically identified (see the “Morphological Identification” section below). Then, more larval habitats were sampled in this locality on November 26th to confirm the presence of the species (Fig. 1C). Moreover, to verify the species’ establishment in this region, the adjacent locality of San Agustín was successfully sampled on November 27th (Fig. 1D). In total, in Rosario del Yata, sampling was conducted in five human dwellings, resulting in the collection of five larval samples in peridomestic containers, while in San Agustín, sampling was conducted in nine human dwellings, resulting in the collection of 14 larval samples in peridomestic containers (Table). The locations of the samples are depicted in Fig. 1C for Rosario del Yata and Fig. 1D for San Agustín. Specific characteristics of the samples are detailed in Table. In all sampled sites, larvae were reared to the L4 instar and adult stages before preservation, utilising 70% alcohol for larvae and pinning for adults.

**TABLE:** Samples and mosquito species collected in Rosario del Yata and San Agustín

Locality	Sample ID	Type of collection^*a*^	Habitat	Date of collection	Collected species
Rosario del Yata^*b*^	FLX-2023-021^*c*^	L	200 L plastic drum	23/11/2023	*Ae. albopictus* *Ae. aegypti* *Tx. haemorrhoidalis*
Rosario del Yata	FLX-2023-042	L	Used tyre	28/11/2023	*Ae. aegypti*
Rosario del Yata	FLX-2023-043	L	Cut plastic bottle with citric cutting in water	28/11/2023	*Ae. albopictus*
Rosario del Yata	FLX-2023-044A	L	Cut plastic bottle with rain water	28/11/2023	*Ae. albopictus*
Rosario del Yata	FLX-2023-045	L	Tyre animal waterer	28/11/2023	*Cx. coronator*
San Agustín	FLX-2023-032	L	100 L concrete animal waterer	27/11/2023	*Cx. coronator* *Cx. quinquefasciatus* *Cx. corniger*
San Agustín	FLX-2023-032B	L	Cut plastic bottle	27/11/2023	*Cx. quinquefasciatus*
San Agustín	FLX-2023-032C	L	Aluminum pot	27/11/2023	*Ae. aegypti*
San Agustín	FLX-2023-033A	L	200 L plastic drum	27/11/2023	*Ae. aegypti*
San Agustín	FLX-2023-033B	L	Plastic bucket	27/11/2023	*Ae. albopictus* *Ae. aegypti* *Tx. haemorrhoidalis*
San Agustín	FLX-2023-033C	L	10 L plastic canister	27/11/2023	*Ae. albopictus* *Ae. aegypti* *Cx. declarator*
San Agustín	FLX-2023-034	L	Used tyre	27/11/2023	*Cx. declarator*
San Agustín	FLX-2023-035	L	1000 L plastic drum	27/11/2023	*Ae. fluviatilis* *Cx. quinquefasciatus*
San Agustín	FLX-2023-036	L	200 L plastic drum	27/11/2023	*Ae. fluviatilis* *Cx. quinquefasciatus*
San Agustín	FLX-2023-037	L	200 L plastic drum	27/11/2023	*Ae. fluviatilis* *Cx. quinquefasciatus*
San Agustín	FLX-2023-038A	L	Plastic bathtub	27/11/2023	*Cx. quinquefasciatus*
San Agustín	FLX-2023-038B	L	Tire animal waterer	27/11/2023	*Ae. fluviatilis*
San Agustín	FLX-2023-039	L	Tire animal waterer	27/11/2023	*Ae. fluviatilis* *Ps. albigenu*
San Agustín	FLX-2023-040	L	30 L plastic bucket	27/11/2023	*Ae. albopictus* *Ae. aegypti*
Rosario del Yata	FLX-2023-044B	A	Collected while biting us at FLX-2023-044A	28/11/2023	*Ae. albopictus* *Ma. titillans*

*a*: type of collection: L = larvae, A = adults; *b*: locality where *Aedes albopictus* was first discovered; *c*: first record of *Ae. albopictus*.

**Fig. 3:**
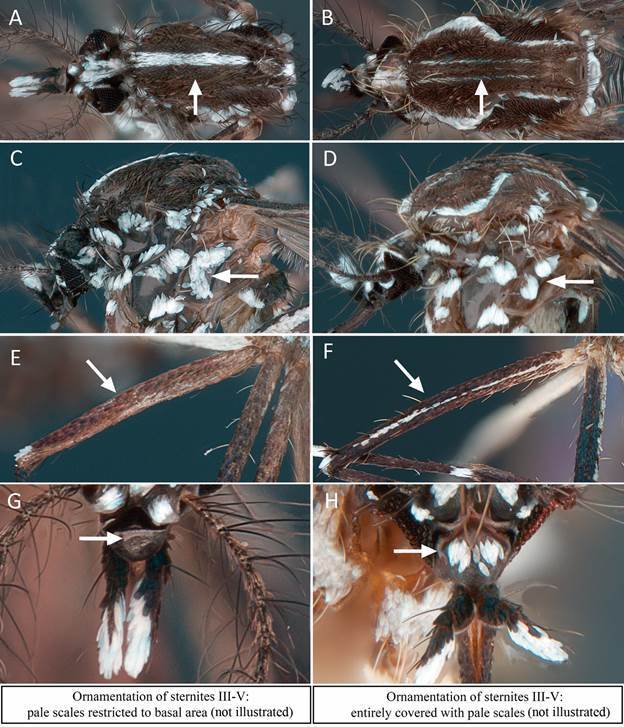
morphological characteristic used to distinguish between *Aedes albopictus* and *Aedes aegypti* in adults. Specimens are from sample FLX-2023-021 of Rosario del Yata. Ornamentation of the scutum: narrow white median longitudinal stripe against a black background in *Ae. albopictus* (A), and lyre-shaped silvery white stripes and narrow submedian longitudinal white stripes against a black background in *Ae. aegypti* (B). Ornamentation of the mesepimeron: two white scale spots connected in a unique V shape spot in *Ae. albopictus* (C), and two white scale spots well separated in *Ae. aegypti* (D). Ornamentation of midfemur (II): pale scales restricted to the basal area in *Ae. albopictus* (E), and entirely covered with pale scales in *Ae. aegypti* (F). Ornamentation of clypeus: without silver scales, entirely black in *Ae. albopictus* (G), and silvery white scales in one or two patches in *Ae. aegypti* (H).

**Fig. 4:**
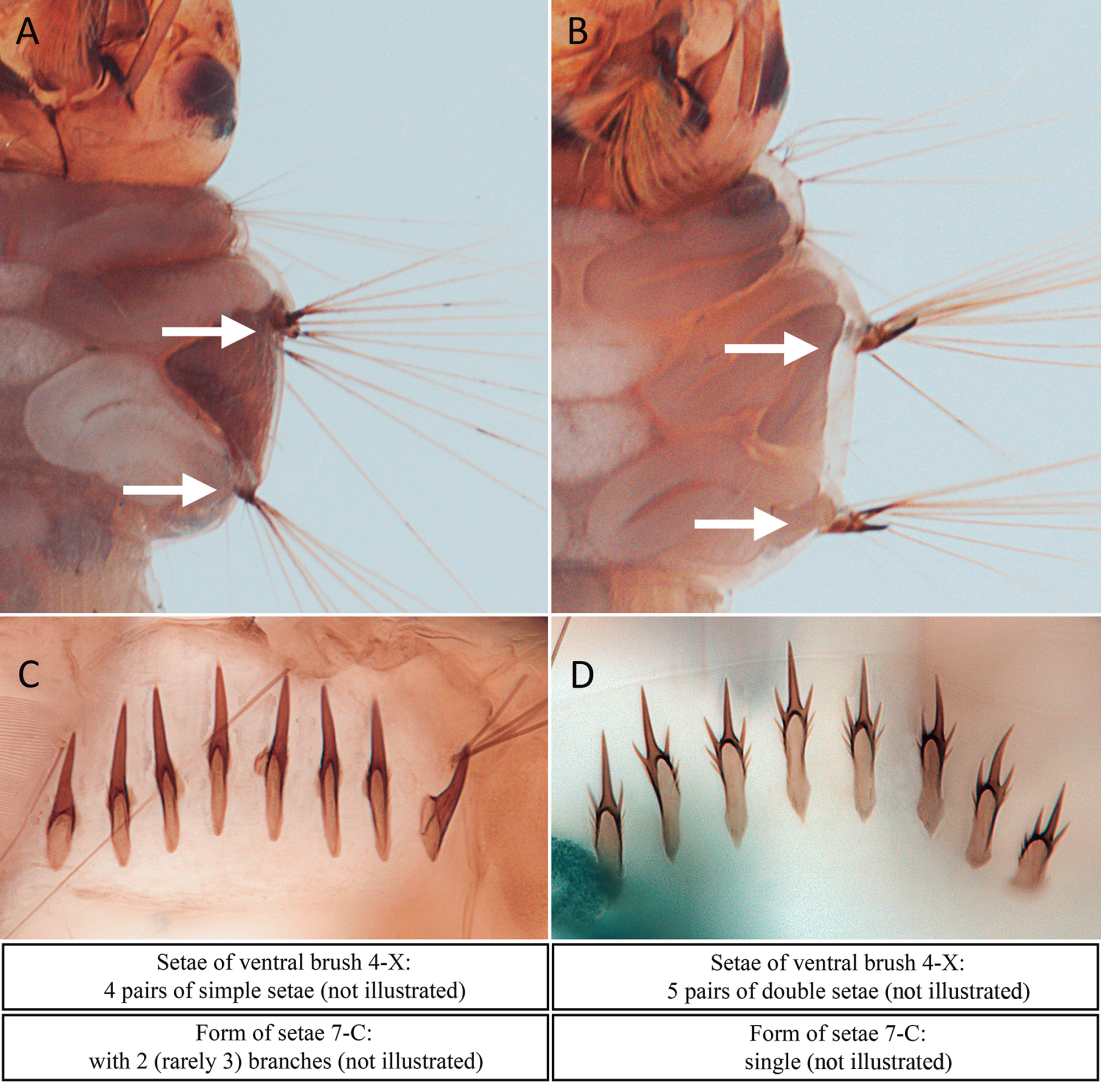
morphological characteristic used to distinguish between *Aedes albopictus* and *Aedes aegypti* in L4 larvae. Specimens are from sample FLX-2023-021 of Rosario del Yata. Ornamentation of lateral support plates of setae 11-M and 11-T of meso- and metathorax: with tiny small spines in *Ae. albopictus* (A), and with large well-marked curved spines in *Ae. aegypti* (B)*.* Form of the median comb scale of segment VIII: spine very long and sharp, with tiny lateral spines at the base of the scale in *Ae. albopictus* (C), and strong submedian spines (trident shape) in *Ae. aegypti* (D)*.*

*Species morphological identification* - *Aedes albo-pictus* was morphologically identified and differentiated from the other unique *Stegomyia* species of Bolivia, *Aedes (Stegomyia) aegypti* (Linnaeus in Hasselquist, 1762), using standard identification keys^17^ ^,^ ^18^ ^,^ ^19^ ^,^ ^20^ and principles or other descriptions,^21^ ^,^ ^22^ ^,^ ^23^ ^,^ ^24^ from which distinctive diagnostic characters were extracted for adults (Fig. 3) and larvae (Fig. 4). Other species were identified using general keys^18^ ^,^ ^25^ and principles or other descriptions of the species.

*Molecular identification of Ae. albopictus* - The morphological identification of *Ae. albopictus* was supplemented with molecular analysis using two mitochondrial gene fragments: Cytochrome C oxidase subunit 1 (COX1, also known as COI) and Cytochrome B (CYTB). Three larvae from Rosario del Yata (from samples FLX-2023-021, FLX-2023-043 and FLX-2023-044), and three from San Agustín (from samples FLX-2023-033B, FLX-2023-033C and FLX-2023-040) were used.

DNA extraction followed a protocol previously proposed,^26^ based on CTAB/chloroform/isopropanol chemistry. Primers for both gene fragments were designed by one of us (CB). For COX1: Albo_Cox1_F: 5’- ACA AAT CAT AAA GAT ATT GGA ACA-3’ and Albo_Cox1_R: 5’- AAC TTC TGG ATG ACC AAA AA-3’; and for CYTB: Albo_CYTB_F: 5’- TCA GCC TGA AAT TTT GGA-3’ and Albo_CYTB_R: 5’- CAG GTT GAA TAT CAG GAG T -3’. Polymerase chain reaction (PCR) reactions were performed in 25 µL final volume including 2 µL of DNA (diluted at 20 ng/µL), 1x buffer B (Hot FIREPol^®^), 1.5 mM of MgCL_2_ (Hot FIREPol^®^), 0.8 mM of dNTP (20 mM) (Eurogentec), 5 pmol of each primer and 1 Unit of FIREPol Taq DNA polymerase (Hot FIREPol^®^). PCR amplifications were carried out in a Vapo Protect Thermocycler^®^. Cycling conditions were an initial denaturation at 95ºC for 15 min, followed by 35 cycles of 15 s denaturation at 94ºC, 30 s annealing at 50ºC for COX1 primers and 30 s annealing at 53ºC for CYTB primers and 1 min extension at 72ºC for both primers, and a final extension step at 72ºC for 5 min. The amplicons were sequenced by Azenta services, and the resulting sequences were manually corrected from the Sanger-type chromatograms. Subsequently, they were compared to deposited sequences in GenBank at NCBI using the basic local alignment search tool (BLAST) algorithm. Sequences returning a query coverage of > 98% were selected to ensure removal of excessively short reference sequences. These selected sequences were then parsed using VSEARCH clustering commands^27^ to identify all closely related haplotypes to our own sequences. Subsequently, maximum likelihood trees (ML trees) were constructed using the MEGA7 program^28^ employing the best model of multiple substitutions suited to the data. The robustness of the clustering was assessed via a bootstrap procedure consisting of 100 replicates.^29^

*Data preservation and storage* - In the field, essential ecological data including GPS coordinates, types of larval habitats, and water quality were collected and organised using a cellular phone equipped with the VECTOBOL database (https://vectobol.ird.fr). The database was updated whenever an internet connection was available. The database was designed with REDCap electronic data capture tools hosted at IRD-France.^30^ ^,^ ^31^ Then, in the laboratory, once species identifications were completed for each sample, the database was updated.

All collected samples have been deposited in the collections of the Medical Entomology Laboratory (LEMUMSS) at the Universidad Mayor de San Simón in Cochabamba, Bolivia. Some vouchers of *Ae. albopictus* were also deposited in the Entomology Laboratory of the Instituto Nacional de Laboratorios de Salud (INLASA) of the Ministry of Health in La Paz, Bolivia. In both collections, the sample’s IDs (which also correspond to the specimen prefix in collection) align with those listed in Table.

The DNA sequences of *Ae. albopictus* were deposited in GenBank with numbers PP465543 to PP465547 for COX1 and PP471874 to PP471878 for CYTB.

## RESULTS

*Collected species* - In the two sampled localities, *Ae. albopictus* was present in six of the 14 sampled houses, three in Rosario del Yata and three in San Agustín. The survey resulted in the identification of a total of 19 positive larval habitats among which six were positive for *Ae. albopictus* (Fig. 2C-D, Table). During the survey, one *Ae. albopictus* female (along with one *Mansonia titillans*) was captured while biting one of our team members (RTL) during larvae collection at sample ID: FLX-2023-044B. Apart from *Ae. albopictus*, eight other mosquito species were collected from larval habitats. They were: *Ae. aegypti*, *Ae. fluviatilis*, *Culex corniger*, *Cx. coronator*, *Cx. declarator*, *Cx. quinquefasciatus*, *Psorophora albigenu* and *Toxorhynchites haemorrhoidalis*. As far as species associations are concerned, *Ae. albopictus* was collected alone in two instances, while in four instances, it was found in association with *Ae. aegypti* either alone, or also with *Cx. declarator*, or *Tx. haemorrhoidalis* (Table).

**Fig. 5:**
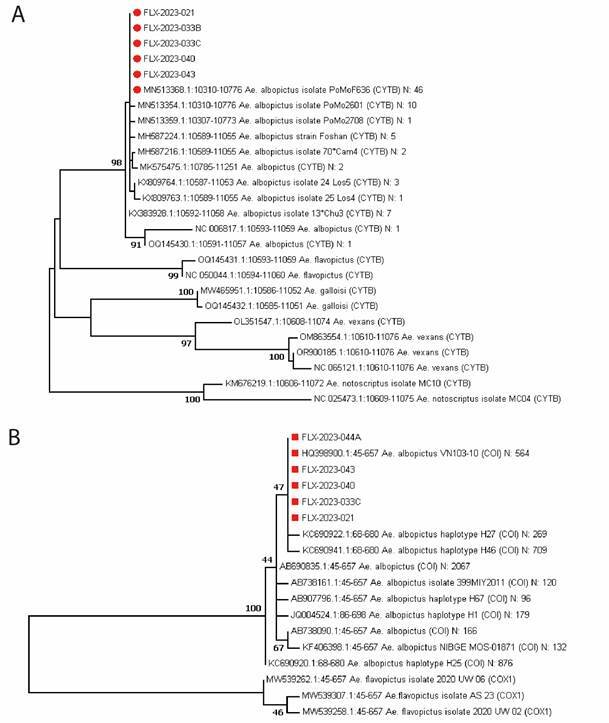
maximum likelihood (ML) tree of 26 different CYTB haplotypes constructed from an alignment of 467 nucleotide positions (A). ML tree of 18 different COX1 (also called *COI*) haplotypes constructed from an alignment of 613 nucleotide positions (B). In both figures, the numbers at the nodes correspond to the bootstrap values > 70%. The best multiple substitution model was the Tamura-Nei model +G (Gamma distribution). Our five isolates exactly matching with the GenBank reference MN513368.1 and HQ398900.1 for CYTB and COX1 respectively are visualised as red dots.

*Molecular identification of Ae. albopictus* - The morphological identifications of *Ae. albopictus* were strongly supported by the molecular results. Specifically, for CYTB, the sequences obtained from five isolates precisely matched 46 reference sequences in GenBank, including accession number MN513368.1, as depicted in the ML tree (Fig. 5A). Notably, this particular CYTB haplotype was detected across various countries such as Albania, Brazil, China, France, Greece, Italy, Japan, Portugal, and the United States. The tree analysis revealed *Ae. flavopictus* as the most closely related sister clade to *Ae. albopictus*.

Regarding COX1 (also known as COI), the sequences obtained from five isolates also exhibited an exact match with 564 reference sequences in GenBank, including accession number HQ398900.1, visualised in the ML tree (Fig. 5B). In line with CYTB, this specific COX1 haplotype was recorded in multiple countries, including Brazil, where infested regions border Bolivia. This new ML tree further confirmed *Ae. flavopictus* as the most closely related species to *Ae. albopictus.*

## DISCUSSION

The current study definitively identifies *Ae. albopictus* in diverse locations within Rosario del Yata and San Agustín, employing both standard morphological characteristics and molecular biology (COX1 and CYTB sequencing). In the field, more than 300 *Ae. albopictus* larvae were collected of which more than 100 were reared to the adult stage. Moreover, one adult female was captured by chance in the field. This discovery, duly documented, puts an end to anecdotes that reported the species without substantiating evidence, a factor that has persistently generated doubt in numerous scientific articles.

In this study, *Ae. albopictus* was collected from artificial larval habitats characteristic of peridomestic environments. In some cases, it coexisted with *Ae. aegypti* and other species that inhabit similar conditions, forming species assemblages similar to those found in nearby geographical areas.^32^

The source of infestation in Rosario del Yata and San Agustín remains elusive. The presence of widespread haplotypes across both gene fragments precluded the identification of a potential origin for the initial insects colonising the area. While sequencing additional genes might provide insight, the identical haplotypes observed in Brazil for both COX1 and CYTB suggest a plausible Brazilian origin for these insects as the most parsimonious hypothesis. It’s noteworthy that molecular identification effectively distinguishes *Ae. albopictus* from *Ae. flavopictus*, a closely related species that can be morphologically confused with *Ae. albopictus*.^33^

The time of arrival is also unknown. However, it may be close to the current discovery date as predicted by a model suggesting a timeframe of 3 to 5 years from 2023 in the event of introduction via the tire or plant trade.^34^ The recent introduction hypothesis is further supported by the inability to find *Ae. albopictus* in the country, as indicated by numerous studies on *Ae. aegypti* which, since 2005, have consistently failed to report the collection of *Ae. albopictus*. This is despite the potential cohabitation of these two species in larval habitats and the expectation of finding the latter species.^35^ ^-^ ^41^ Furthermore, in the three databases, (1) the GBIF database, encompassing 90,413 worldwide references,^42^ (2) the WRBU-VectorMap database and (3) the “*Global compendium of Aedes aegypti and Ae. albopictus occurrence*”,^4^ ^,^ ^43^ all lack records of *Ae. albopictus* occurrences in Bolivia but present collection points for *Ae. aegypti*. Bibliographic searches using standard databases (Web of Science, PubMed, bioRxiv) do not reveal any documented occurrences of the species in Bolivia. Two of the article authors (FL and PB) did not find *Ae. albopictus* in 2022 during a similar entomological survey carried out in southern Beni, although they easily collected *Ae. aegypti* in the Municipality of Palos Blancos, San Borja, Trinidad, Ascensión de Guarayos and Okinawa Uno. During the present 2023 survey, excluding the localities of Rosario del Yata and San Agustín, *Ae. albopictus* was not found, while *Ae. aegypti* was readily captured in the cities of Guayaramerin, Cachuela Esperanza and Inicua, suggesting a higher occurrence of the latter and, consequently, a more recent establishment of *Ae. albopictus*. In fact, before this study, there is no document or voucher conclusively demonstrating the presence of *Ae. albopictus* in the country.^6^

*Aedes albopictus* displays phenotypic plasticity, enabling the development of strains adapted to a broad range of environmental conditions and optimal temperatures for survival and development.^44^ ^,^ ^45^ The mosquito has been collected along altitudinal transects, reaching up to 2100 m above sea level in Nepal,^46^ and is commonly found above 1200 m in tropical environments.^47^ ^,^ ^48^ With a demonstrated capacity to outcompete *Ae. aegypti* in diverse ecological contexts^49^ and given the detection of *Ae. aegypti* in Bolivia’s major cities, including cities above 2000 m such as Sucre at 2700 m^50^ and Cochabamba at 2600 m,^51^ it is probable that *Ae. albopictus* could eventually establish itself in all these urban areas.

*In conclusion* - When established in a new habitat, *Ae. albopictus* becomes of significant epidemiological concern, serving as a confirmed vector for the chikungunya virus and dengue virus, among others. The multifaceted topography and climate of Bolivia give rise to discrete ecosystems that intricately shape the prevalence and distribution of diverse insect vectors. Given the likelihood of *Ae. albopictus* colonising regions where *Ae. aegypti* is already present, covering approximately two-thirds of the territory below 2,700 metres in altitude, it becomes imperative for the Health Services overseeing vector surveillance to swiftly develop a situational map of *Ae. albopictus* occurrence in the country. This includes monitoring the species colonisation process and incorporating *Ae. albopictus* into the targeted species for control measures. This proactive approach is crucial for effective public health management and the prevention of potential disease outbreaks associated with this invasive mosquito species.
